# Survey mode and asking about future intentions did not impact self-reported colorectal cancer screening accuracy

**DOI:** 10.1186/1471-2288-14-19

**Published:** 2014-02-05

**Authors:** Timothy J Beebe, Jeanette Y Ziegenfuss, Sarah M Jenkins, Kandace A Lackore, Timothy P Johnson

**Affiliations:** 1 Survey Research Center, Division of Health Care Policy and Research, Department of Health Sciences Research, Mayo Clinic, 200 First Street SW, Rochester, MN, USA; 2 Health Partners Institute for Education and Research, Health Partners, Mail Stop 21111R, PO Box 1524, Minneapolis, MN, USA; 3 Division of Biomedical Statistics and Informatics, Department of Health Sciences Research, Mayo Clinic, 200 First Street SW, Rochester, MN, USA; 4 Survey Research Laboratory, University of Illinois at Chicago, 629 CUPPA Hall, 412 South Peoria, Suite 115, Chicago, IL, USA

**Keywords:** Data collection, survey methodology, cancer screening, self-report accuracy, mail surveys, telephone surveys

## Abstract

**Background:**

Self-reported colorectal cancer (CRC) screening behavior is often subject to over-reporting bias. We examined how the inclusion of a future intention to screen item (*viz*. asking about future intentions to get screened before asking about past screening) and mode of survey administration impacted the accuracy of self-reported CRC screening.

**Methods:**

The target population was men and women between 49 and 85 years of age who lived in Olmsted County, MN, for at least 10 years at the time of the study. Eligible residents were randomized into four groups representing the presence or absence the future intention to screen item in the questionnaire and administration mode (mail vs. telephone). A total of 3,638 cases were available for analysis with 914, 838, 956, and 930 in the mail/future intention, mail/no future intention, telephone/future intention, and telephone/no future intention conditions, respectively. False positives were defined as self-reporting being screened among those with no documented history of screening in medical records and false negatives as not self-reporting screening among those with history of screening.

**Results:**

Comparing false positive and false negative reporting rates for each specific screening test among the responders at the bivariate level, regardless of mode, there were no statistically significant differences by the presence or absence of a preceding future intention question. When considering all tests combined, the percentage of false negatives within the telephone mode was slightly higher for those with the future intention question (6.7% vs 4.2%, p = 0.04). Multivariate models that considered the independent impact of the future intention question and mode, affirmed the results observed at the bivariate level. However, individuals in the telephone arm (compared to mail) were slightly (though not significantly) more likely to report a false positive (36.4% vs 31.8%, OR = 1.11, p = 0.55).

**Conclusion:**

It may be that in the context of a questionnaire that is clearly focused on CRC and with specific descriptions of the various CRC screening tests, certain design features such as including intention to screen items or mode of administration will have very little impact on the accuracy of self-reported CRC screening.

## Background

Large sample surveys such as the Health Information National Trends Survey (HINTS), the Behavioral Risk Factor Surveillance System (BRFSS) Survey, and National Health Information Survey (NHIS) routinely collect self-reported colorectal cancer (CRC) screening information, but the accuracy of the information coming from these important sources remains largely unknown as the scant literature on the accuracy of self-reported CRC screening is equivocal. While some have found consistent over-reporting of CRC screening relative to various criterion or gold standards such as medical records [1,2], some have found such self-reports to be quite accurate [3,4]. A recent meta-analysis conducted by Rauscher and colleagues found that nearly half of self-reported positive CRC screening histories were likely to be negative in administrative or medical records; however, they also found that under-reporting was of concern [5].

There is evidence that questionnaire design features such as item wording and context, as well as mode of data collection (e.g., mail or telephone), can impact the accuracy of all self-reported health behaviors in general and cancer screening behavior in particular [1,6]. One particular questionnaire design feature, asking about future intentions to get screened before asking about past screening behavior, has been found to increase the accuracy of the latter when compared to medical records, possibly because respondents are under less social pressure to over-report past practice of that behavior [6]. In our own research in this area, we found that asking about future intentions to get screened before asking about past CRC screening behavior, significantly lowered reports of past CRC screening and that the effect of the positioning of the future intentions item varied by survey mode [7]. Specifically, in the mailed survey, the odds of reporting past CRC screening were almost three times greater in the condition where we asked about future intentions *after* the CRC screening question (“future second”) as compared to the condition where we asked about intentions first (“future first”); in the telephone condition, the odds of reporting were only 28% higher in the “future second” condition than in the “future first” condition. The results suggest that asking about future intentions to get screened before the actual behavior elicits lower - and arguably more truthful - reports of CRC screening, but only in mailed surveys.

Although this prior work was among the first to test the effect of asking about future intentions and mode of administration simultaneously in a factorial design and with a community sample (as opposed to less generalizable patient samples), the study had some potentially important limitations. First, whereas past investigations have focused on consistency between self-reports and medical records as the primary measure of accuracy, we only looked at the former in the initial 2008 study as we did not have medical record data with which to compare. We have addressed this limitation by focusing on consistency between CRC self-reports and medical records as the primary measure of accuracy in the present investigation. Second, the earlier study utilized items that did not include complete descriptions of the screening tests, contrary to the recommendations of some [3,8]. The absence of such descriptions may have led to confusion among respondents as to what the screening tests were and, thus, limited the inferential value of our findings. To address this limitation, the present study utilized items more aligned with the manner of question asking in major surveys such as the BRFSS and HINTS, which include detailed descriptions of each of the CRC tests.

In the current paper, we retain the community and methodological elements of our prior work but focus on consistency between self-reports and medical records as the primary measure of accuracy, drawing on the strengths of the Rochester Epidemiology Project (REP) - the medical record linkage system for health care providers to residents of Olmsted County (home of the Mayo Clinic and Olmsted Medical Center). We also utilize items more aligned with the manner of question asking in major surveys (e.g., BRFSS and HINTS) that have been tested for reliability and validity. By deploying a more optimal design in the present investigation, we are in a better position to inform the collection of self-reported CRC screening rates in surveys such as the NHIS, BRFSS, and HINTS than we were after the prior investigation.

## Methods

### Study setting and population

The investigation described herein uses data collected from residents of Olmsted County, Minnesota. With the exception of a higher proportion of the working population employed in the health care industry, population characteristics of Olmsted County are similar to those of the US white population [9]. The Rochester Epidemiology Project (REP), which is the medical record linkage system for health care providers to residents of Olmsted County, served as the sample frame for the study. The REP chronicles the medical care delivered to community residents from the early 1900’s to the present. The REP captures and classifies diagnostic and procedural information from these records, including hospitalizations, office visits, emergency room visits, and nursing home care. The REP enables the conduct of population-based studies by affording access to patients’ medical history from all medical care providers of residents of Rochester and Olmsted County, Minnesota, at Mayo Clinic and the other area medical care facilities [10]. Annually, over 80% of the entire population is attended by one or more of the facilities included in the REP, and 96% are seen at least once during any given four-year period [11]. Therefore, the REP medical records linkage system provides what is essentially an enumeration of the geographically defined population from which samples can be drawn.

The target population was men and women between 49 and 85 years of age who lived in Olmsted County for at least 10 years at the time the sample was drawn in order to minimize the opportunities for screening outside of the REP catchment area. Eligibility status was determined from the REP medical record and administrative data. Potential participants were excluded if there was a diagnosis of colon cancer or a history of genetic syndromes.

### Study design

The primary objective of this research was to test the unique and interactive effects of asking about future intention to get screened for CRC (either before the past CRC screening behavior question or not at all) and survey mode (mail versus telephone) on the accuracy of self-reported CRC screening behavior. The study design used permitted us to assess negative, as well as positive, CRC screening histories and, therefore, the degree of under- and over-reporting across question order and survey mode. Both those with NO history of any CRC screening in the REP and those WITH a history of at least one CRC screening in the REP were purposively sampled at an approximate 2:1 ratio and randomly assigned to the mail or telephone modes. CRC screening tests included fecal occult blood test (FOBT), sigmoidoscopy, colonoscopy, computerized tomographic colonography (CT colonography), and barium enema. Eligible participants were then randomly assigned to one of two question wording conditions within mode where they received the future intention to get screened for CRC either before the CRC screening item or not at all (described below).

### Self-report questionnaire

We modified the format of the NCI CRCS questionnaire developed by Vernon and colleagues that provides CRC screening test descriptions prior to asking questions about test use [8]. For each of the five CRC screening tests, respondents were read (or in the mail version instructed to read) a test description and then asked whether they had ever had it, heard of it, reason for exam, and facility location (Mayo Clinic, Olmsted Medical Center, or elsewhere). Participants randomly assigned to the condition that included the future intentions question received a version of the NCI CRCS questionnaire that included that item (“Are you planning on being tested for [CRC screening test] in the next 12 months?”) just after the description of a given screening test but prior to the question asking about past screening behavior. Participants who had heard of the test and had had it were also asked to report whether it occurred within defined time intervals that were held constant across screening test type: 1) No test; 2) A year ago or less; 3) More than 1 but not more than 2 years ago; 4) More than 2 but not more than 5 years ago; and 5) More than 5 years ago. Those in the condition without the future intentions question received only the descriptions of the screening tests and the questions asking about past screening behavior. Questions about socio-demographics (race/ethnicity, education, and marital status) and family history of colorectal cancer were also included as part of the questionnaire for all respondents. All versions of the questionnaire were subjected to formal pretests conducted prior to data collection. The precise wording of all study items is available from the authors.

### Survey data collection process

Survey data collection was performed by the Mayo Clinic Survey Research Center (SRC) between November 2010 and April 2012. A multiple contact data collection protocol was deployed for both mail and telephone conditions per the method proposed by Dillman [12]. For those in the mail survey mode, the initial mailing packet consisted of a cover letter, two HIPAA Authorization Forms (one to be returned and one for the participant’s files) that granted permission for the researchers to link surveys with medical record information from the REP, a survey, and a business reply envelope. A reminder postcard was mailed approximately ten days after the initial mailing. A third mailing was sent to survey non-respondents approximately two weeks after the mailing of the postcard reminder.

For those randomly assigned to the telephone mode, the interviewer first completed the interview and then indicated that in order to use the supplied interview data, the respondent would have to fill out a HIPAA Authorization Form (HAF). If the respondent indicated that he or she did not have a copy of the HAF available from the prior mailing, another one was sent the same day of the interview. Calls to attempt an interview were made by trained CRC interviewers at a variety of times of the day (morning, afternoon, and evening) and days of the week (weekdays and weekends). Telephone numbers were attempted up to five times per case and messages were left on answering machines to increase participation. At any point, if a subject expressed a desire not to participate, he or she was not contacted further. All consent and study procedures were approved by the Institutional Review Boards at Mayo Clinic and Olmsted Medical Center.

A total of 6,023 and 3,396 eligible participants were assigned to the mail and telephone modes, respectively, and invited to complete the survey. The response proportion was calculated as the number of completions divided by the number of eligible participants using the response rate calculation formula (RR3) set forth by the American Association for Public Opinion Research [13]. A total of 1,752 mailed surveys were received with a completed HAF, for an overall response rate of 29% (1,752 of 6,023). A total of 1,886 telephone interviews were completed – and a signed HAF returned - for an AAPOR response rate of 56% (1,886 of 3,396). A total of 3,638 cases were available for analysis with 914, 838, 956, and 930 in the mail/future intention, mail/no future intention item, telephone/future intention, and telephone/no future intention item conditions, respectively.

### Statistical analysis

Survey respondents were compared between the four conditions with respect to the sociodemographic information we had available in the REP. These analyses enabled assessment of selection into mode based on differential mode preference after random assignment and to identify any possible confounders that warranted adjustment in the primary analyses. Response rates were compared between modes with a chi-square test. Overall comparisons were performed with chi-square tests (gender and race) and analysis of variance (age). Further, the distribution within each condition was compared to the entire group (total eligible) using chi-square goodness-of-fit tests for gender and race, and with one-sample t-tests for age, treating the overall distribution as fixed. To determine whether false self-reports of each (FOBT, sigmoidoscopy, colonoscopy, CT colonography, and barium enema) or any (any one of those listed) past CRC screening behavior varied by condition, the self-reported percentages were compared by mode and presence or absence of the future intention question using chi-square tests (or Fisher’s exact test, as appropriate). To evaluate the accuracy of self-reported CRC screening and using information in the REP as the standard, false positives were defined as self-reporting being screened among those with no documented history of screening. False negatives were defined as not self-reporting screening among those with history of screening. The “truth” with respect to history of screening was defined by whether or not there was evidence of past CRC screening in the REP at any time prior to the fielding of the survey or self-reported screening outside of Olmsted County (which would not be available in the REP).

Based on the premise that respondents tend to over-report socially desirable health behaviors such as CRC screening and that our manipulations were most likely to impact false positives, logistic regression analysis was used to determine whether or not false positive rates varied by mode of data collection, the presence or absence of the future intentions item, and/or their interaction, adjusting for age, gender, and race. All P values are two sided, and a P value of ≤ 0.05 was regarded as statistically significant. All reported percentages, means, and regression analyses were done using SAS v. 9.3 software (SAS Institute, Inc.).

## Results

Table 1 shows the demographic composition of the respondents by experimental arm. Gender, mean age, and race all varied significantly (P ≤ 0.01) across conditions. These underlying differences necessitated the use of an adjusted analysis described later, to fully disentangle the impact of the future intention questions.

**Table 1:** Responder demographics by experimental arm

	**Mail**^**†**^		**Phone**^**†**^			
	**Intention (N = 2248)**	**No intention (N = 3775)**	**Intention (N = 1701)**	**No intention (N = 1695)**	**Total Eligible (N = 9419)**	**P value**^**‡**^
Responders, N (%)	914 (40.7%)	838 (22.2%)	956 (56.4%)	930 (54.9%)	3638 (38.6%)	< 0.0001
Among Responders						
Gender, N (%)		**	**	**		***
F	503 (55.0%)	390 (46.5%)	572 (59.8%)	531 (57.2%)	4947 (52.5%)	
M	411 (45.0%)	448 (53.5%)	384 (40.2%)	398 (42.8%)	4470 (47.5%)	
Age	**	**				***
Mean (SD)	65.2 (10.2)	66.4 (10.8)	63.1 (10.0)	63.7 (10.3)	63.2 (10.4)	
Race, N (%)	**	**	**	**		*
Caucasian	874 (95.6%)	795 (94.9%)	907 (94.9%)	889 (95.6%)	8677 (92.1%)	
Other	21 (2.3%)	11 (1.3%)	31 (3.2%)	22 (2.4%)	504 (5.4%)	
Unknown	19 (2.1%)	32 (3.8%)	18 (1.9%)	19 (2.0%)	238 (2.5%)	

*0.01 ≤ P-value < 0.05.

**P-value < 0.01.

***P-value < 0.001.

^†^P-values (denoted by asterisks) are comparing distributions of responders within each mode to the total eligible (treating “Total” as fixed).

^‡^Overall p-value comparing the distributions of responders between the four conditions.

Among the screening tests considered, the most common were sigmoidoscopy and colonoscopy. Documented screening rates were significantly higher among responders as compared to non-responders within the mail (86.2% vs 62.1%) and telephone (75.2% vs 55.4%) modes (p < 0.0001 for each). Comparing false positive and false negative reporting rates for each specific screening test among the responders at the bivariate level, within mode (mail or telephone), there were no statistically significant differences by the presence or absence of a preceding future intention question (Table 2). When considering all tests combined, however, the percentage of false negatives within the telephone mode was slightly higher for those with the future intention question (6.7% vs 4.2%, p = 0.04). Although the false positive rates were slightly higher for those with the future intention question (mail: 34.2% vs 29.5%; phone: 38.1% vs 34.8%), these differences were not statistically significant.

**Table 2:** **Inaccurate self**-**reports by screening test among respondents**

	**Mail**			**Phone**		
	**Intention**	**No intention**	**P-value**^**††**^	**Intention**	**No intention**	**P-value**^**††**^
All responders, N	914	838		956	930	
FOBT, N with/without documented test	76/838	84/754		62/894	73/857	
n (%) false negatives	47 (61.8%)	62 (73.8%)	0.10	37 (59.7%)	37 (50.7%)	0.30
n (%) false positives	168 (20.1%)	141 (18.7%)	0.50	186 (20.8%)	179 (20.9%)	0.97
Sigmoidoscopy, N with/without documented test	449/465	426/412		373/583	356/574	
n (%) false negatives	159 (35.4%)	147 (34.5%)	0.78	152 (40.8%)	142 (39.9%)	0.81
n (%) false positives	74 (15.9%)	76 (18.5%)	0.32	63 (10.8%)	72 (12.5%)	0.36
Colonoscopy, N with/without documented test	755/159	660/178		691/265	648/282	
n (%) false negatives	111 (14.7%)	92 (13.9%)	0.68	89 (12.9%)	85 (13.1%)	0.90
n (%) false positives	30 (18.9%)	27 (15.2%)	0.37	60 (22.6%)	51 (18.1%)	0.19
CT colonography, N with/without documented test	30/884	21/817		24/932	25/905	
n (%) false negatives	6 (20.0%)	10 (47.6%)	0.064*	4 (16.7%)	11 (44.0%)	0.062*
n (%) false positives	60 (6.8%)	44 (5.4%)	0.23	31 (3.3%)	32 (3.5%)	0.80
Barium enema, N with/without documented test	25/889	15 / 823		22/934	14/916	
n (%) false negatives	5 (20.0%)	1 (6.67%)	0.38*	0 (0%)	0 (0%)	--
n (%) false positives	286 (32.2%)	268 (32.6%)	0.86	220 (23.6%)	221 (24.1%)	0.77
*Any screening*^†^, N with/without documented test	794/120	716/122		733/223	686/244	
n (%) false negatives	36 (4.5%)	33 (4.6%)	0.94	49 (6.7%)	29 (4.2%)	0.04
n (%) false positives	41 (34.2%)	36 (29.5%)	0.44	85 (38.1%)	85 (34.8%)	0.46

*Fisher’s Exact.

^†^“Any screening” was defined as whether or not the person had any of the specific tests in the table.

^††^P-values for comparison of false negatives between intention vs no intention, and for comparison of false positives between intention vs no intention.

Among the 3638 respondents, 709 had no documented history of screening. Of these 247 (34.8%) self-reported screening (“false positives”). Table 3 provides the results of the logistic regression analyses adjusting for age, race (Caucasian vs non-Caucasian), and gender where we ran a series of models for the false positive outcome, focusing on the group with no documented history of screening. Individuals in the phone arm (compared to mail) were slightly (though not significantly) more likely to report a false positive (36.4% vs 31.8%, OR = 1.11, p = 0.55). In these multivariate models, we observed no independent impact of the future intention question on accuracy in either mode (36.7% with intention item vs 33.1%, OR = 1.18, p = 0.31). Simultaneously considering mode and future intention, we observed that the effects of the telephone mode (vs. mail) and future intention question were virtually unchanged (OR = 1.12 and 1.18, respectively). In a model that included an interaction between future intention and mode, the odds ratio for future intention was 1.04 for mail and 1.26 for phone, suggesting that the effect may have been stronger in the phone mode, however, this was not statistically significant (p-value for interaction = 0.58). Of note, neither gender nor race was significantly associated with false positive rates in any of the models considered. Although females were slightly less likely to provide false self-reports, this was not statistically significant (in each model: OR = 0.74, p = 0.07). There was a statistically significant effect of age on false positive rate. Older individuals were more likely to falsely report a screening test (in each model: OR = 1.04 for 1-year increase in age, p < 0.0001).

**Table 3:** **Multivariable analysis predicting probability of false positive self**-**report of any screening among responders with no history of screening**

	**Unadjusted**		**Adjusted**^**†**^	
	**OR**	**95% CI**	**OR**	**95% CI**
Model 1				
Phone vs mail	1.23	0.89, 1.71	1.11	0.79, 1.56
Model 2				
Future intention item present vs not present	1.17	0.86, 1.60	1.18	0.86, 1.62
Model 3^‡^				
Phone vs mail	1.24	0.89, 1.72	1.12	0.79, 1.57
Future intention item present vs not present	1.18	0.86, 1.60	1.18	0.86, 1.62

^†^Adjusted for age, race (Caucasian vs other), and gender.

^‡^In an adjusted model that included an interaction between future intention and mode, the odds ratio for future intention was 1.04 for mail and 1.26 for phone, suggesting that the effect may have been stronger in the phone mode, however this was not statistically significant (p-value for interaction = 0.58).

## Discussion

Going into the present investigation, we had hypothesized that asking about future intentions to get screened for CRC before the question about past screening behavior would increase the accuracy of the latter when compared to medical records. In our earlier study, we found that asking about intention before screening resulted in lower reports of past screening by a rather significant amount [7]. Similarly, Johnson and colleagues [6] found that reports of Pap testing and mammography were more accurate when a screening intention item was placed before the questions regarding past screening. The current results show very little impact of the intention item on CRC reporting accuracy and the observed pattern of results suggest that its inclusion may actually *decrease* accuracy when compared to medical records.

Why our results run counter to what has been observed in the literature is unclear, but it could be due to the role social desirability may or may not play in the accuracy of self-reported CRC screening. The impact of the intention item is premised on the notion that by asking people if they intend to engage in a socially desirable behavior such as CRC screening *before* the actual past behavior, they will feel less pressure to misrepresent their past practice of that behavior in a positive light. However, recent studies have found that the accuracy of self-reported CRC screening is unrelated to social desirability [14,15], so one could posit that the accuracy of CRC screening would be little impacted by the inclusion of the intention item.

Why our current results differ so significantly from our own work may be due to the methodological limitations of the latter listed in the introduction, primarily the absence of definitional clarity. It may be that in the context of a questionnaire with specific descriptions of the various CRC screening tests as recommended by Vernon and colleagues [8], certain design features such as including intention items - or even mode of administration - will have very little impact. Alternatively, the discordance in results observed between our two studies may be due to the varied composition of the responding samples. Whereas, the data collection platform in our earlier study was an “omnibus” survey whereby CRC screening items were one among many other types of questions being asked (e.g., general health, health insurance coverage, use of online health information, attitudes towards tattoos, attitudes towards drug use, oral health, and attitudes towards surveys), the present survey focused solely on CRC and was titled as such. Having the survey content so manifest in the present study might have brought in a different type of respondent than a survey so broadly cast as our prior omnibus survey. As respondents to CRC surveys have been shown to be more likely to have had an updated CRC screening test than non-respondents [16] – something we observed in the present investigation as well - differential selection of respondents represents a plausible explanation for the different findings.

Although self-reported CRC screening accuracy was not associated with the inclusion of the intention item in our study, it is worth noting that accuracy tended to be higher in the mail administration mode rather than telephone, albeit not statistically significantly. This is consistent with the meta-analysis of the extant research on CRC screening accuracy conducted by Rauscher and colleagues [5] who found that compared to self-administered modes of data collection, telephone interviews tended to be less accurate in terms of sensitivity, specificity, and positive predictive value. Those same authors noted that the number of studies available for inclusion in their analysis was small and recommended further work in this area. In our prior study, we found no statistically significant main effect of mode, although the results indicated higher reports in the mailed survey condition [7]. We are in the process of analyzing the impact of mode on the accuracy of specific types of CRC screening in the present study - including the emergent technology of CT Colonography - as some have noted differential impact of administration mode across the various CRC screening tests [17].

## Conclusion

In conclusion, our experimental findings extend the work on self-reported CRC screening accuracy, generally, and the impact of certain survey design features such as questionnaire layout, item wording, and mode of administration in particular. We found little impact of the intention item and suggestive evidence mailed surveys positively affect accuracy. Future research should continue to identify mechanisms underlying the over-reporting of positive health behaviors, such as CRC screening, and do so with more heterogeneous populations than the one of focus in the present investigation. However, it is possible that CRC screening is not viewed as a positive health behavior along the lines of healthy dieting or exercise and the literature associated with those types of behavior is not germane or translatable to CRC screening. As such, other factors contributing to the over-reports observed with this behavior beyond social desirability need to be explored. Our findings also underscore the importance of offering definitional clarity when asking about complex and potentially confusing cancer screening tests, such as those included in the CRC realm, as well as other areas such as Pap test and mammography. Given the importance of clear and defensible estimates of cancer screening to clinical and health policy experts, coupled with the seemingly contradictory findings to date, continued dedication to finding the optimal manner in which to secure such estimates is paramount.

## Competing interests

The author’s declare that they have no competing interests.

## Authors’ contributions

TB, JZ, and TJ made substantial contributions to the conception and design of the study and acquisition of the data. They also, along with SJ and KL, made substantial contributions to the analysis and interpretation of the data. All authors contributed to the drafting of the manuscript, reviewed it, and made important suggested revisions. All authors also approved the final, submitted version of the manuscript. The manuscript has not been published (in full or in part) either in print or on-line and is not under consideration for publication elsewhere.

## Pre-publication history

The pre-publication history for this paper can be accessed here:

http://www.biomedcentral.com/1471-2288/14/19/prepub
